# Pb Induces MCP-1 in the Choroid Plexus

**DOI:** 10.3390/biology11020308

**Published:** 2022-02-15

**Authors:** Huiying Gu, Yundan Xu, Nicole Du, Yongqi Yu, Wei Zheng, Yansheng Du

**Affiliations:** 1 Department of Neurology, Indiana University School of Medicine, Indianapolis, IN 46202, USA; huiygu@iupui.edu (H.G.); yundanxu@163.com (Y.X.); yoyu@iupui.edu (Y.Y.); 2 School of Basic Medical Science, Hubei University of Chinese Medicine, Wuhan 430065, China; 3 Department of Pediatrics, Children’s National Hospital, Washington, DC 20010, USA; ndu@childrensnational.org; 4 School of Health Sciences, Purdue University, West Lafayette, IN 47907, USA; wzheng@purdue.edu

**Keywords:** lead, Alzheimer’s disease, MCP-1, choroid plexus, Z310, macrophage infiltration, p38 MAP kinase, NF-κB

## Abstract

**Simple Summary:**

Our data in this study show that oral Pb exposure can induce MCP-1 expression along with monocyte and macrophage infiltration in the choroid plexus. Both NF-κB and p38 MAPK pathways modulate Pb-induced MCP-1 expression in CP epithelial cells.

**Abstract:**

Lead (Pb) is an environmental element that has been implicated in the development of dementia and Alzheimer’s disease (AD). Additionally, innate immune activation contributes to AD pathophysiology. However, the mechanisms involved remain poorly understood. The choroid plexus (CP) is not only the site of cerebrospinal fluid (CSF) production, but also an important location for communication between the circulation and the CSF. In this study, we investigated the involvement of the CP during Pb exposure by evaluating the expression of the monocyte chemoattractant protein-1 (MCP-1). MCP-1 is highly expressed in the CP compared to other CNS tissues. MCP-1 regulates macrophage infiltration and is upregulated in AD brains. Our study revealed that Pb exposure stimulated MCP-1 expression, along with a significantly increased macrophage infiltration into the CP. By using cultured Z310 rat CP cells, Pb exposure stimulated MCP-1 expression in a dose-related fashion and markedly activated both NF-κB and p38 MAP kinase. Interestingly, both SB 203580, a p38 inhibitor, and BAY 11-7082, an NF-κB p65 inhibitor, significantly blocked Pb-induced MCP-1 expression. However, SB203580 did not directly inhibit NF-κB p65 phosphorylation. In conclusion, Pb exposure stimulates MCP-1 expression via the p38 and NF-κB p65 pathways along with macrophage infiltration into the CP.

## 1. Introduction

The demand for Pb in industry has been steadily increasing during the last decade. In addition to the occupational hazard, environmental exposure to Pb continues to be a major public health concern, as Pb is widely present in the air, drinking water, household products, plastics, and painted materials [1]. While Pb can cause acute toxicity, previous human studies suggested that cumulative lifetime Pb exposure is also associated with accelerated declines in cognition and dementia [2]. Workers exposed to Pb show brain atrophy and behavioral deficits [3,4,5]. A follow-up study examining Pb workers revealed that cumulative Pb-dose-related exposure is associated with progressive declines in cognitive function as well as alterations in brain structure [2]. Additionally, reports show the presence of higher levels of Pb in diffuse neurofibrillary tangles in AD cases compared to control individuals [6,7]. A retrospective human study demonstrated a relationship between prenatal Pb exposure and the alteration of genes and enzymes implicated in AD senile amyloid plaque formation [8]. Cumulative evidence shows Pb exposure induces both amyloid deposition and tau hyperphosphorylation in animal brains [9,10,11,12]. However, despite these findings, it is currently unclear how Pb contributes to the pathogenesis and pathology of AD and related dementia (ADRD). Understanding Pb exposure and its relationship to ADRD is of urgent importance for the broader community. Data from the United States show that those exposed to high levels of Pb in the 1960s and 1970s are now at an age where they are at higher risk of developing AD, with an estimated prevalence of 8.4–13.8 million [13,14].

Monocyte chemoattractant protein-1 (MCP-1), also referred to as C–C motif chemokine ligand 2 (CCL2), is a chemoattractant or chemokine that is expressed in a variety of cells including endothelial cells, smooth muscle cells, fibroblasts, epithelial cells, mesangial cells, astrocytes, myeloid cells, T cells, and tumor cells. After binding to its receptor, CC-chemokine receptor 2 (CCR2), this chemokine is a potent chemoattractant for the infiltration and migration of immune cells to sites of inflammation, including monocytes, natural killer cells, memory T cells, and immature dendritic cells [15]. Additionally, MCP-1 directly activates or primes monocytes and macrophages to produce a variety of inflammatory cytokines, and manages cell adhesion and chemotaxis by regulating integrin expression and localization [16]. The MCP-1–CCR2 signaling axis is implicated in many inflammatory and neurodegenerative diseases such as atherosclerosis, multiple sclerosis, asthma, neuropathic pain, diabetic nephropathy, and cancer [17,18]. Recently, MCP-1 and CCR2 were also reported to be closely associated with decline in memory over time in a symptomless aging group [19]. Increased MCP-1 levels are also related to the pathogenesis of Alzheimer’s disease (AD) [20,21]. In addition to increased MCP-1 expression observed in the brain tissue [22], both serum and cerebrospinal fluid (CSF) levels of MCP-1 are significantly elevated in patients with mild cognitive impairment (MCI) and mild AD [23,24,25]. Higher CSF MCP-1 levels are associated with disease progression in AD [26,27] and increased levels of tau and its phosphorylation in the CSF of elderly symptomless people [28]. Therefore, the MCP-1–CCR2 signaling axis is a potential target for the treatment of inflammation-related diseases such as AD.

The choroid plexus (CP), which contains a monolayer of CP epithelial cells, is the epithelial bilayer blood–CSF barrier (BCB) in the lateral and fourth ventricles. In contrast to the blood–brain barrier (BBB), which is formed by endothelial cells lining the continuous capillaries, CP epithelial cells are located on top of fenestrated capillaries. Both the CP epithelial cells at the BCB and endothelial cells at the BBB are interconnected with tight junctions that regulate the transport of molecules and pathogens between blood and the brain. The BCB, together with the BBB, protects the brain from detrimental effects of peripheral blood, enables entry of various necessary molecules through the entire CNS, and facilitates the clearance of waste and toxins. Although both barriers have similar functions, they differ with regard to their morphologic and functional properties. The CP–CSF system plays a pivotal role in brain development, maintenance of brain homeostasis and function, and the repair process [29]. Another major task of the CP epithelial cells is the production and secretory function of CSF and its contained molecules [30]. Importantly, the CP was shown to be a key selective gateway that governs immune cell recruitment and infiltration into the brain [31] as well as regulation of cytokine and chemokine levels in CSF [32]. A disruption of the tight junction architecture and dysregulation of transporters in the CP contribute to the pathophysiology of many neurological and neuropsychiatric diseases [33]. Interestingly, BCB dysfunction is present in AD patients [34], and this was further confirmed by showing decreased expression of tight junction components in diseased CP endothelial cells [35]. In animal studies, it has been demonstrated that CP epithelial cells and their contained transporters play important roles in the regulation of Aβ and tau clearance from CSF to blood [30]. It has also been observed that the CP is involved in AD pathogenesis [36,37]. Interestingly, compared to other brain domains, MCP-1 in the CP is highly expressed and may be an important factor involved in dysregulation of CP function in brain diseases [32].

Human BCB was demonstrated as a specific target of Pb toxicity [38]. Additionally, Pb, MCP-1, and BCB all are involved in AD development, although their relationship remains unknown. In the present study, we sought to examine whether Pb exposure mediates the induction of MCP-1 expression in the CP and what molecular mechanisms underly this induction by using both in vivo and in vitro approaches.

## 2. Materials and Methods

### 2.1. Animals and Treatment

Tg-SwDI (APP-Swedish, Dutch, Iowa) mice were introduced from the Jackson Laboratory (Bar Harbor, ME, USA) and bred in the laboratory of Animal Center at the Indiana University School of Medicine (Indianapolis, IN, USA). All procedures and materials used in this animal study were approved by the Institutional Animal Care and Use Committee (IACUC) at the Indiana University School of Medicine. Five-month-old female mice were housed, 3 to 5 each cage, supplied ad libitum with food and water, in a 12 h light/dark cycle facility. In this study, three mice orally received 27 mg Pb/kg (i.e., 50 mg Pb-acetate/kg) (Sigma-Aldrich Corporation, St. Louis, MO, USA) daily by gavage (Pb-treated group). The control group orally received an equivalent molar concentration of Na-acetate daily [39]. All mice were sacrificed at 30 days after the first oral treatment.

### 2.2. Immunohistochemistry and Quantitation of Macrophage and MCP-1, Phosphorylated NF-kB p65- and p38-Positive CP Cells

After perfusion-fixation with 4% paraformaldehyde in 0.1 M phosphate buffer (pH 7.4) and cryoprotection in 30% sucrose/phosphate buffer, the brains were frozen in liquid nitrogen and serially sectioned (30 µm) through the entire choroid plexus. Tissue sections were incubated successively with rat CD45 monoclonal antibody (1:100; ThermoFisher, Hampton, NH, USA), rabbit anti-Iba-1 polyclonal antibody (1:400, ThermoFisher, Hampton, NH, USA), MCP-1, phosphorylated p65, and phosphorylated p38 followed by the anti-rat IgG antibody conjugated with Alexa Fluor 647 (1:500, ThermoFisher, Hampton, NH, USA), anti-rabbit antibody conjugated with FITC (1:500; ThermoFisher, Hampton, NH, USA), or anti-mouse antibody conjugated with FITC (1:500; ThermoFisher, Hamton, NH, USA). To quantify Iba1+/CD45+ double positive macrophages in the CP, we utilized the nuclear counterstain with DAPI (Vector Laboratories, Burlingame, CA, USA). Iba1+/CD45+ double-positive macrophages were counted in 9 serial sections, spaced 210 μm apart. The stereological method for counting macrophages vs. cells was applied as previously described [10]. Similarly, without counterstaining with DAPI, we quantified the green fluorescence intensity of MCP-1, phosphorylated NF-κB p65, and phosphorylated p38 MAP kinase in the CP.

### 2.3. Cultures of Choroidal Epithelial Z310 Cells

The Z310 rat immortalized choroidal epithelial cell line was developed with characteristics, culture, and maintenance procedures of this cell line described in a previous publication [40]. Cells were maintained and used as previously described in our laboratory [41]. In brief, the cells were cultured in Dulbecco’s modified eagle medium (DMEM) supplemented with 10% fetal bovine serum (FBS), 10 ng/mL epidermal growth factor (EGF), 100 U/mL of penicillin, 100 mg/mL of streptomycin, and 40 mg/mL of gentamycin in a humidified incubator with 95% air and 5% CO_2_ at 37 °C and passaged twice a week for two weeks. During experiments, cells were plated first in 24-well plates at a density of 5 × 10^5^ cells/mL. Cultures were used 2 days after preparation. Autoclaved 5 mM PbAc stocking solution was prepared by dissolving PbAc in sterile double-deionized water. The cells were pretreated with or without inhibitors for 1 h, followed by adding 1–5 µM Pb treatments for an additional 24 h to determine the release and expression of MCP-1. At the end of the treatments, both cell-free media and cells were collected for further protein analyses.

### 2.4. Western Blot Analysis

Western blot analysis was performed on cell extracts [10] that were prepared by lysing cells in a buffer containing 1% Nonidet P-40, 0.1% SDS, 50 mM Tris (pH 8.0), 50 mM NaCl, 0.05% deoxycholate, and protease inhibitor (Roche Diagnostics Corporation, Indianapolis, IN, USA). After proteins in cell extracts were determined by using a Bradford assay kit according to the manufacture’s instruction, 100 µg/lane of proteins was size-fractionated on a 4–12% polyacrylamide gradient gel (SDS-NuPAGE) and transferred onto nitrocellulose (Hybond N; Amersham Biosciences, Piscataway, NJ, USA). Blots were then blocked in 5% skim milk for 2 h, and incubated at 4 °C overnight with primary antibodies against phospho-p38 (1:500, Cell Signaling, Danvers, MA, USA); p-38 (1:1000, Danvers, MA, USA); phospho-NF-κB (1:500, Cell Signaling, Danvers, MA, USA); NF-κB (1:1000, Danvers, MA, USA); phospho-ATF-2 (1:500, Cell Signaling, Danvers, MA, USA); ATF-2 (1:1000, Cell Signaling, Danvers, MA, USA); phospho-IKKα (1:500, Cell Signaling, Danvers, MA, USA); IKKα (1:1000, Cell Signaling, Danvers, MA, USA). After washing, blots were incubated with IRDye 800CW anti-rabbit or IRDye 680RD anti-mouse secondary antibodies (1:5000, Licor, Lincoln, NE, USA) and imaged with an Odyssey Imaging System (Licor, Lincoln, NE, USA). The optical density (OD) of the blot band intensity was further quantified using Image J [42] and reported in the OD ratio of phosphorylated protein/total protein.

### 2.5. MCP-1 ELISA

After 1 h pre-incubation with/without 10 µM SB 203580 or 1 µM BAY 11-7082, Z310 cells were treated with 1 µM Pb for an additional 24 h. Cell-free media and cells were then collected. After cells were lysed, MCP-1 levels in Z310 cells and cell-free media were measured using an MCP1 Rat ELISA Kit (R&D SYSTEMS, Minneapolis, MN, USA) following the manufacturer’s instructions. Additionally, total proteins in cell extracts were also determined by using a Bradford assay kit according to the manufacture’s instruction.

### 2.6. Statistical Analysis

Statistical analyses were performed by a one-way ANOVA with post hoc comparisons by the Dunnett’s test. All data from different experimental groups are expressed as mean ± SD. Differences between means of two groups were considered significant when *p* < 0.05.

## 3. Results

### 3.1. Pb Exposure Induced MCP-1 Expression and Enhanced Macrophage Infiltration in the CP Tissues

**Figure 1:**
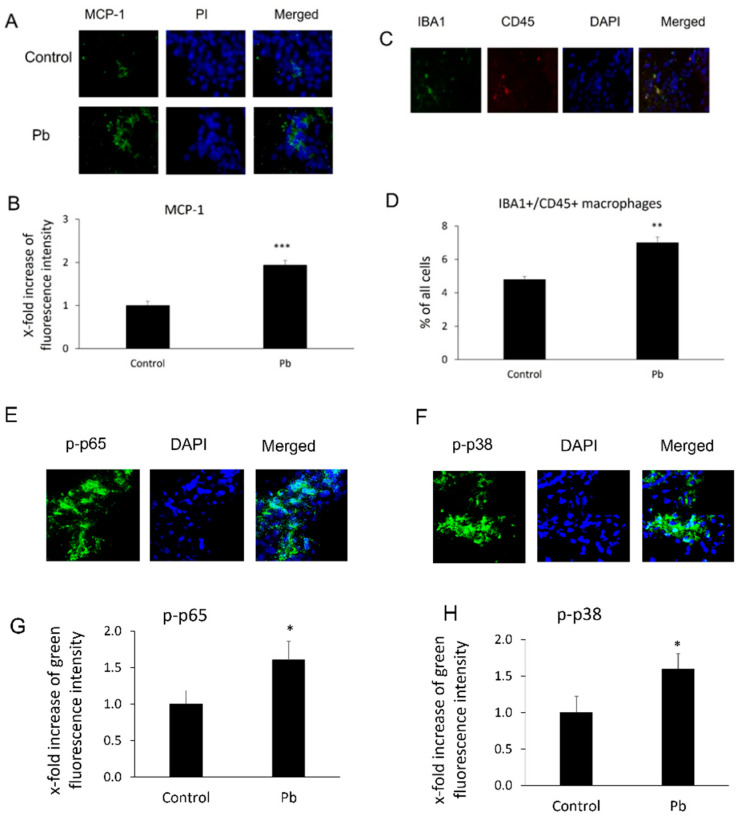
Pb exposure induced MCP-1 expression, phosphorylated NF-κB p65 (p-p65), phosphorylated p38 MAPK (p38), and macrophage infiltration in the CP tissues. Tg-SwDI transgenic mice received oral gavage of 50 mg/kg Pb acetate once daily for 30 days. At the end of Pb exposure, mice brain sections were stained with antibodies of MCP-1, p-p65, and p-p38 to determine MCP-1 expression and levels of p-p65 or p-p38, as well as with both Iba1 and CD45 antibodies to detect macrophage infiltration in the CP. (**A**) Representative MCP-1 immunofluorescent images used for counting. (**B**) Quantification of MCP-1 expression by analyzing total MCP-1 fluorescent intensity in the CP. Upregulation of MCP-1 expression in Pb-treated mice was observed compared to that in mice without Pb treatments in the CP. (**C**) Representative double immunofluorescent images of Iba1+/CD45+ used for counting. (**D**) Quantification of Iba1+/CD45+ macrophages in the CP. (**E**) Representative p-p65 immunofluorescent images used for counting. (**F**) Quantification of p-p65 levels by analyzing total p-p65 fluorescent intensity in the CP. (**G**) Representative p-p38 immunofluorescent images used for counting. (**H**) Quantification of p-p38 levels by analyzing total p-p38 fluorescent intensity in the CP. Upregulation of MCP-1 expression, macrophage infiltration, and p-p65 and p-p38 in Pb-treated mice was observed as compared to mice without Pb treatments in the CP. Data are presented as mean ± SD, *n* = 3/group. * *p* < 0.05, ** *p* < 0.01, *** *p* < 0.001.

After Tg-SwDI mice were treated with or without Pb daily for 30 days, MCP-1 expression levels and macrophage infiltration in mouse CP areas were evaluated by using immunohistochemical assays. As shown in Figure 1A,B, chronic Pb exposure significantly increased expression of MCP-1 in this area. The fluorescence intensity of MCP-1 in the CP increased 1.9-fold compared to the control group (*p* < 0.001). Additionally, since MCP-1 is a chemoattractant for macrophage migration and infiltration, we investigated if induction of MCP-1 was able to affect the macrophage number in the CP. As expected, along with the induction of MCP-1, Pb exposures also increased Iba-1+/CD45+ macrophages in the CP areas. The percentage of Iba1+/CD+ macrophages against total cells markedly increased from 4.8 ± 0.19% to 7.0 ± 0.33% (*p* < 0.01, Figure 1C,D). Both the NF-κB and p38 MAPK pathways were implicated in the induction of MCP-1 expression [43]. We therefore evaluated the fluorescence intensity of phosphorylated NF-κB p65 (p-p65) and phosphorylated p38 MAP kinase (p-p38) in the CP. As expected, either p-p65 or p-p38 MAP kinase increased 1.6-fold (Figure 1E–H) compared to the control group (*p* < 0.05).

### 3.2. Pb Exposure Induced MCP-1 Expression in Rat Choroidal Epithelial Z310 Cells

**Figure 2:**
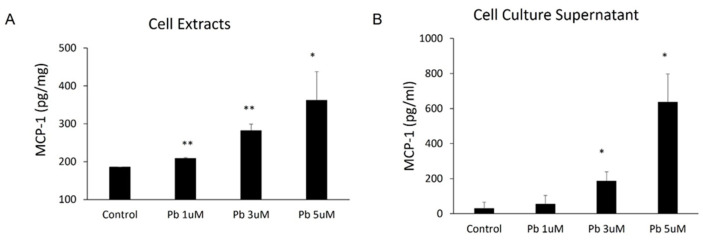
Pb exposure induced MCP-1 expression in rat choroidal epithelial Z310 cells. Z310 cells were treated with 1, 3, or 5 µM Pb for 24 h. MCP-1 levels in both cell extracts (**A**) and cell-free culture supernatants (**B**) were determined by using ELISA. Data represent mean ± SD, *n* = 3/group. *: *p* < 0.05, **: *p* < 0.01.

Since the CP epithelial cells are likely the major source of MCP-1 production in the CP, we employed immortalized choroidal epithelial Z310 cells to further evaluate our in vivo observation that Pb is able to induce MCP-1. As shown in Figure 2, Pb treatments induced MCP-1 expression in a dose-related fashion within 24 h. Pb at 1, 3, and 5 µM markedly induced MCP-1 expression from 185.4 ± 0.58 pg/mg to 208.5 ± 2.14 pg/mg (*p* < 0.01), 281.9 ± 17.19 pg/mg (*p* < 0.01), and 361.7± 75.7 pg/mg (*p* < 0.05) (Figure 2A), respectively. Consistent with the expression data, 1, 3, and 5 µM of Pb also stimulated MCP-1 release into culture media from 30.0 ± 36.06 pg/mL to 55 ± 49.24 pg/mL, 186.7 ± 52.04 pg/mL (*p* < 0.05), and 636.7 ± 160/73 pg/mL (*p* < 0.05), respectively (Figure 2B).

### 3.3. Both NF-κB p65 and p38 MAPkinase Were Activated in Z310 Cells by Pb

**Figure 3:**
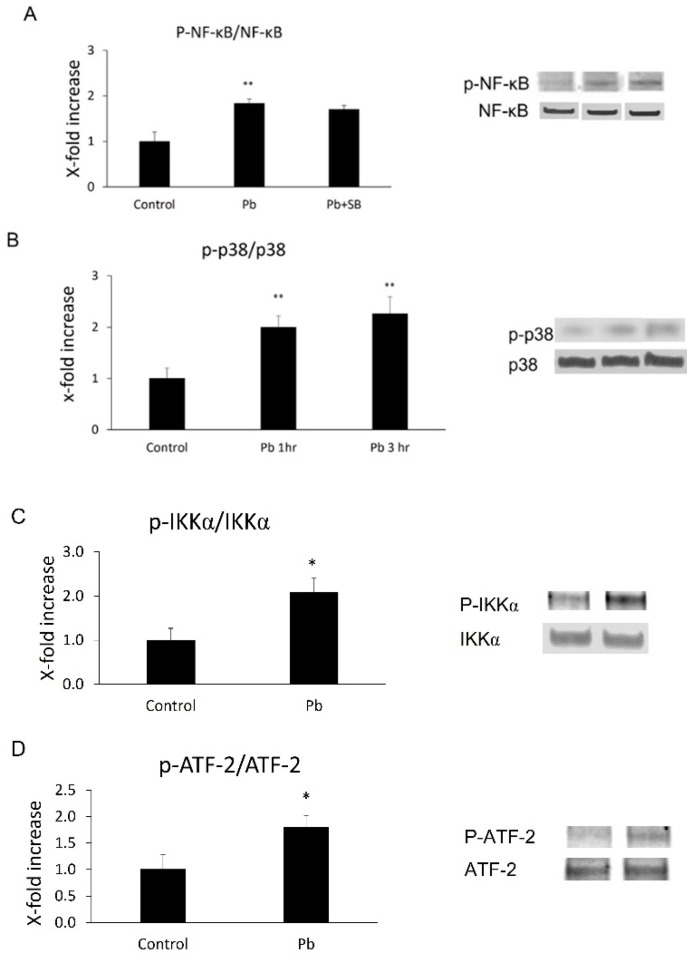
Phosphorylation levels of NF-κB p65, IKKα, p38 MAP kinase, and ATF-2 in Z310 cells followed by Pb treatments were determined by Western blot. (**A**,**C**) After 1 h pre-treatments with or without 10 µM SB 203580, Z310 cells were incubated with 1 µM Pb for an additional 1 h. Cells were then lysed and levels of phosphorylated and total NF-κB p65 (p-p65, p65) or IKKα (p-IKKα and IKKα) in Z310 were determined by Western blot. The phosphorylated protein (p-p65 or p-IKKα) band intensities were quantified and normalized to p65 or IKKα densities using Image J. (**B**,**D**) Z310 cells were treated with 1 µM Pb for 1 or 3 h. Western blot was performed to determine phosphorylated and total p38 MAPK (p-p38, p38) and ATF-2 (p-ATF-2, ATF-2). The phosphorylation levels of p38 MAPK or ATF-2 (p-p38, p-ATF-2) were also normalized by protein levels of total p38 or ATF-2. Data are presented as mean ± SD, *n* = 3/group. *: *p* < 0.05, ** *p* < 0.01.

Based on the in vivo data, we decided to investigate whether Pb exposure is able to induce NF-κB p65 or p38 MAPK activation in CP epithelial cells. Phosphorylation levels of both NF-κB p65 and its upstream factor, IKKα, or p38 MAPK and its downstream substrate, ATF-2, were measured in Z310 cells following 1 or 3 h Pb treatments. As shown in Figure 2, there was a 1.8-fold (*p* < 0.01) increase in phosphor-NF-kB p65 (p-p65) and 2.1-fold (*p* < 0.05) in phosphor-IKKα (p-IKKα) 1 h after Pb exposure (Figure 3A,C). Additionally, there was a 2.0- (1 h after Pb, *p* < 0.01) or 2.3-fold (3 h after Pb, *p* < 0.01) increase in phosphor-p38 MAPK (p-p38, Figure 3B), and a 1.8-fold (3 h after Pb, *p* < 0.05) increase in phosphor-ATF-2 (p-ATF-2, Figure 3D), suggesting Pb exposure is able to activate both pathways in CP epithelial cells.

### 3.4. Both NF-κB and p38 MAPK Inhibitors Blocked Pb-Induced MCP-1 Expression

**Figure 4:**
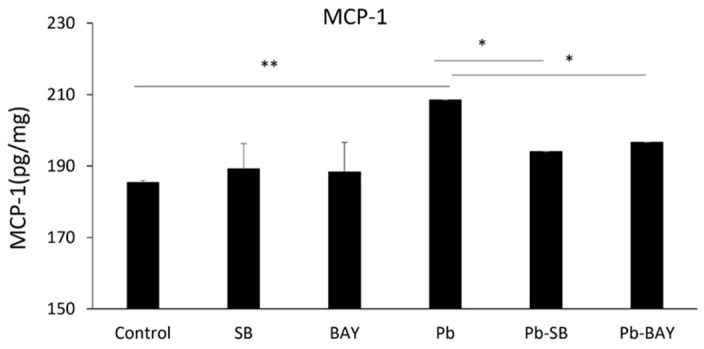
Both NF-κB and p38 MAPK inhibitors blocked Pb-induced MCP-1 expression. Z310 cells were pre-treated with/without 10 µM SB 203580 or 1 µM BAY 11-7082 for 1 h, followed by 1 µM Pb treatments for additional 24 h. Cells were lysed and expression levels of MCP-1 in cell extracts were quantified using ELISA. Data are presented as mean ± SD, *n* = 3/group. * *p* < 0.05, ** *p* < 0.01.

We then investigated whether Pb-induced activations of NF-κB and p38 MAPK underlies Pb-induced MCP-1 expression. As expected, both the NF-κB inhibitor, BAY 11-7082, and the p38 MAPK inhibitor, SB 203580, significantly inhibited Pb-induced MCP-1 expression. MCP-1 expression levels were reduced from 208.5 ± 6.82 pg/mg to 196.6 ± 0.02 pg/mg (*p* < 0.05) by 1 µM BAY 11-7082 and to 194.0 ± 0.03 pg/mg (*p* < 0.05) by 10 µM SB 203580 (Figure 4). Thus, our data show that Pb induces the expression of MCP-1 in the CP epithelial cells via both Pb-induced NF-κB p65 and p38 MAPK pathways.

### 3.5. SB 253,580 Did Not Block NF-κB p65 Phosphorylation

It remains unclear if these two pathways individually or together mediate Pb-induced MCP-1 expression. It was reported that the p38 MAPK pathway does not directly mediate NiCl-induced NF-κB p65 phosphorylation in primary endothelial cells [44] during MCP-1 induction, but targets the downstream factor of NF-κB. Similar to that case, we observed that SB 203580 did not block Pb-induced NF-κB p65 phosphorylation (Figure 3A), suggesting Pb might at least inhibit the downstream target of NF-κB.

## 4. Discussion

In this study, we showed that the CP responded to a 30 day treatment of Pb by elevating the expression of MCP-1 as well as phosphorylation levels of NF-κB p65 and p38 MAP kinase. The elevation in MCP-1 also correlates with Iba-1+/CD45+ macrophage infiltration in the CP. In addition, we demonstrated a possible mechanism of this elevation in MCP-1, showing that Pb-induced MCP-expression in CP epithelial cells is mediated by NF-κB p65 and p38 MAP kinase pathways.

The CP, as an interface between the vascular system and brain parenchyma, is the site of CSF production and performs governance for immune cell recruitment and infiltration into brain. The CP regulates the production of cytokines and chemokines and their levels within CSF [32]. The CP’s role in immunoregulation was found to be related to AD development [45]. Furthermore, this barrier is a clearance gatekeeper influenced by inflammatory factors for efflux of AD risk protein factors such as TREM2, apolipoprotein E, and tau [36]. Interestingly, MCP-1, as a key chemokine regulating inflammatory reactions, is more highly expressed in the CP compared to other brain domains [32]. MCP-1 not only regulates monocyte and macrophage chemotaxis, but also directly stimulates inflammation via cellular and cytokine release. Additionally, MCP-1 influences cell adhesion and blood brain interface permeability by modulating integrin expression and localization [16]. In humans, MCP-1 levels in CSF and blood, regulated by CP, were demonstrated to partially, if not fully, contribute to memory decline in both ageing populations and AD patients. Knockout of MCP-1 demonstrates a markedly beneficial effect in ischemic injury areas and functional recovery [46]. Therefore, MCP-1 in the CP may be the important target to help us understand how Pb is involved in the pathogenesis of AD and other neuronal injury diseases. In this study, by using an AD mouse model, we observed that chronic exposure to Pb stimulates the expression of MCP-1 in the CP and the subsequent migration of macrophages by its chemoattractant function to the same area. Additionally, we showed, for the first time, that Pb exposure directly induces expression and release of MCP-1 in the CP epithelial cells. Our findings thus demonstrate an important pathogenic mechanism by which Pb exposure stimulates CP dysfunction, which is involved in AD pathogenesis. The finding that Pb exposure directly induces MCP-1 expression in CP epithelial cells may partially explain how the CP is involved in Pb-mediated AD pathogenesis.

In order to further confirm and understand the mechanism underlying Pb-induced MCP-1 expression in CP endothelial cells, we employed a widely used Z310 CP epithelial cell line. Accumulating evidence suggests that either the NF-κB or p38 MAPK pathway plays a crucial role in the induction of MCP-1 expression [44]. In this study, we, for the first time, demonstrated that Pb exposure directly induces phosphorylation of NF-κB p65 and p38 MAPK in CP epithelial cells. In order to confirm the role of these two pathways in Pb-induced MCP-1 expression, we used two specific inhibitors of NF-κB and p38 MAPK. Both inhibitors significantly blocked Pb-induced MCP-1 expression, suggesting these two pathways mediate Pb-induced MCP-1 expression. However, since these two pathways regulate different downstream factors, which have different impacts on human health, it is necessary to elucidate if Pb activates these two pathways individually or together regulate MCP-1 production in CP epithelial cells. In particular, NF-κB exerts both beneficial and detrimental effects in AD and other brain diseases [47]. Interestingly, in a previous study, it was demonstrated that NiCl-induced p38 MAPK activity does not directly stimulate NF-κB p65 phosphorylation, but targets the downstream co-factor that contributes to the NF-κB pathway [44]. In our work, similar to that study, SB 203580 also did not directly block Pb-induced p65 phosphorylation. Since p38 MAPK inhibition was proposed as a promising strategy to treat AD [48], our findings suggest that it is possible to use p38 MAPK inhibitors to reduce Pb-induced MCP-1 expression.

Finally, it remains unclear if Pb systemically affects MCP-1 levels, so a further study is underway.

## 5. Conclusions

The data in the present study demonstrate that oral Pb exposure can cause robust induction of epithelial MCP-1 expression along with monocyte and macrophage infiltration into the choroid plexus. Additionally, Pb induced NF-kB and p38 MAP pathways to mediate MCP-1 induction in the CP epithelial cells. The findings suggest a novel pathogenic mechanism involved in Pb-mediated AD pathogenesis.

## Data Availability

Not applicable.
